# Supplementary material for: AraC Functional Suppressors of Mutations in the C-Terminal Domain of the RpoA Subunit of the Escherichia coli RNA Polymerase
Source: Microorganisms. 2024 Sep 23;12(9):1928. doi: 10.3390/microorganisms12091928 (PMC11434276; doi:10.3390/microorganisms12091928)
Supplement: Supplementary file 1 [file microorganisms-12-01928-s001.zip › microorganisms-3207806-supplementary.pdf]

Supplementary FigureS1

C

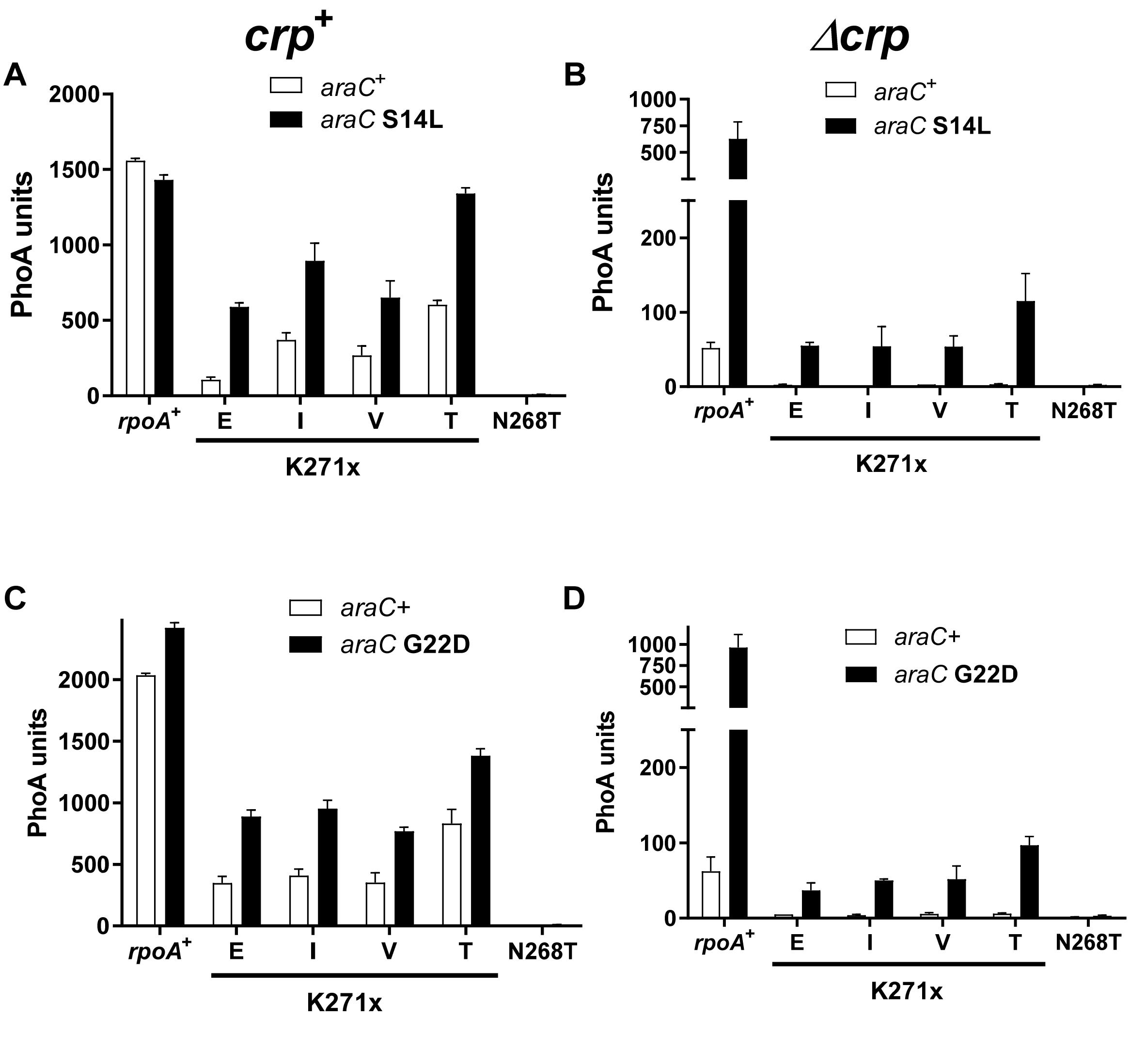

Figure legend. P_{BAD} activity mediated by wild type AraC$^+$ and S14L (A & B) or G22D (C & D). Plasmids expressing AraC$^+$ or the indicated AraC suppressor proteins were introduced in *crp*$^+$ (A & C) or Δ*crp* (B & D) strains with the indicated *rpoA* alleles. Cultures were assayed after a 1h induction with arabinose.

Supplementary Figure S2

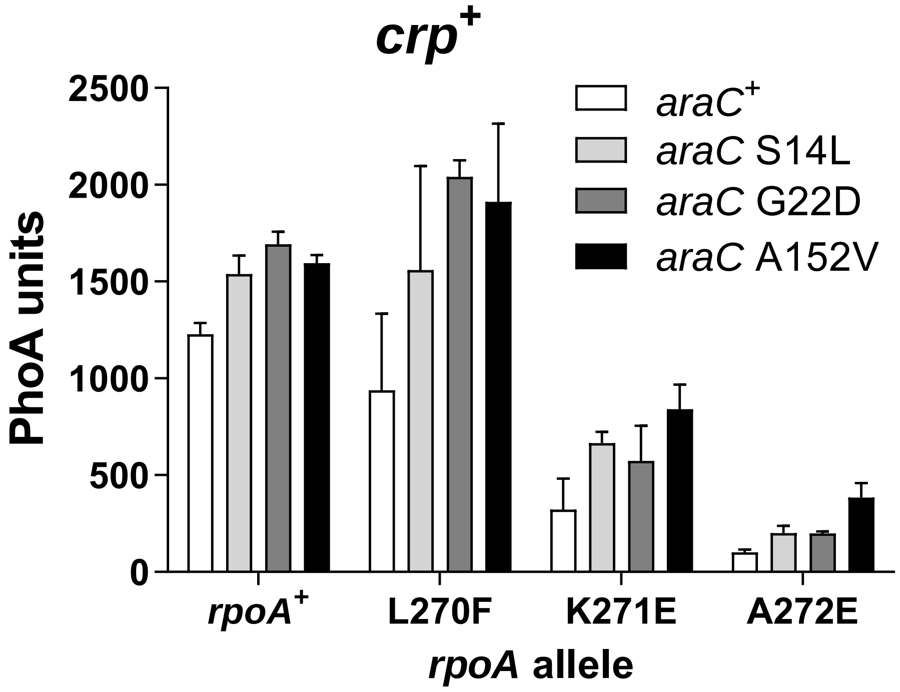

Figure legend. Effects of the AraC suppressors on the two residues that flank K271 of the RpoA α–CTD. Plasmids expressing AraC$^+$ or the indicated AraC suppressor proteins were introduced in *crp*$^+$ strains carrying the *rpoA*$^+$ or the three indicated *rpoA* alleles. Cultures were assayed after a 1h induction with arabinose.

Supplementary figure S3

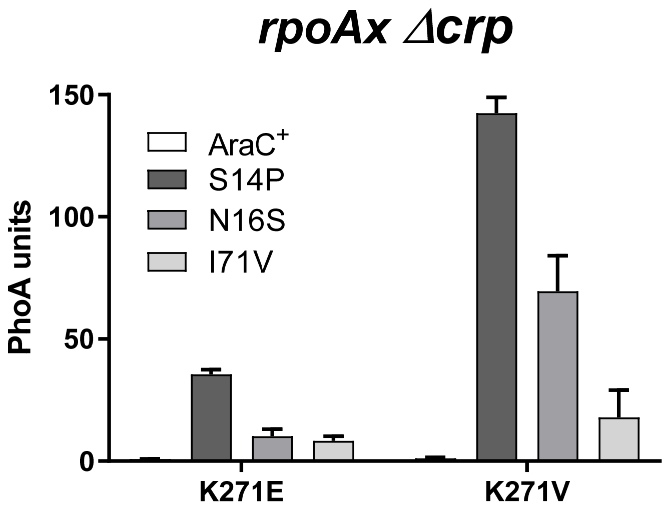

Figure legend. P_{BAD} activity mediated by *araC* suppressors of RpoA A272E. Plasmids expressing $AraC^+$ or the indicated AraC suppressor proteins were introduced in Δ*crp* strains expressing either the RpoA K271E or the K271V proteins. Cultures were assayed after a 1h induction with arabinose.

Supplementary Figure S4

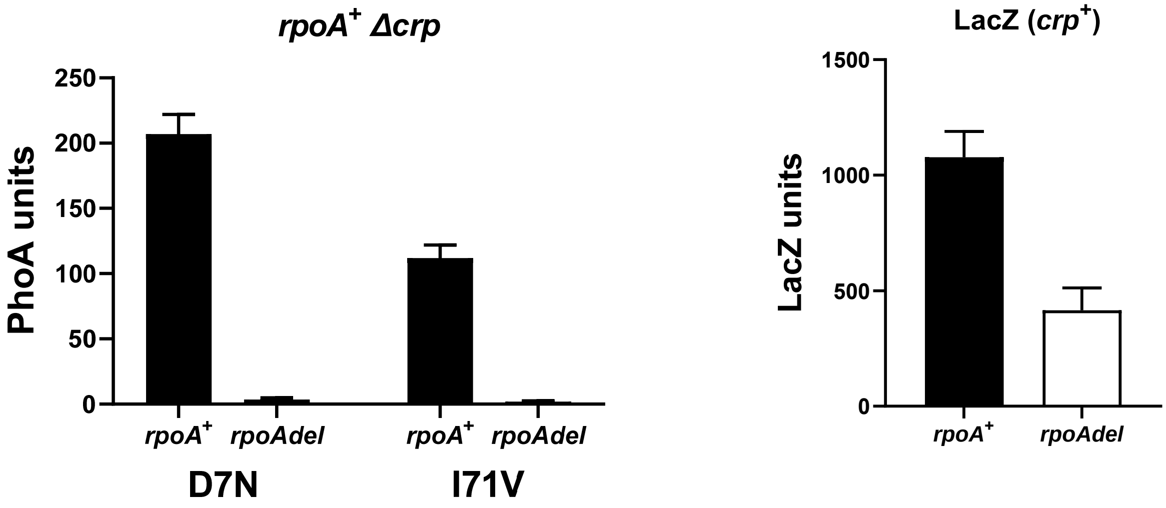

Figure legend. High expression of the *rpoA* α-CTD deletion strongly decreased expression from the *araBAD* promoter. RpoA and the RpoA α-CTD deletion were expressed from the bacteriophage T4 *32* promoter of pRDB8 (Duvoisin et al., 1986). Black bars: pRDB8-*rpoA*$^+$; white bars: pRDB8-*rpoA*delCTD. PhoA expression was measured in a *rpoA*$^+$ Δ*crp* strain. With AraC$^+$, the expression of PhoA was barely detectable with both plasmids.The expression of *lacZ* was measured in the HfrH strain.
